# A visualization study of anterior transpedicular cortical screw placement in the lower cervicalspine

**DOI:** 10.3389/fsurg.2026.1771508

**Published:** 2026-03-23

**Authors:** Chuan He, Xiaolin Li, Changhua Peng, Zexin Hou, Hongwei Chen, Leilei Wu, Ke Wang, Qiusen Deng, Kaijia Xu

**Affiliations:** 1 Jingzhou Hospital of Traditional Chinese Medicine, Jingzhou, Hubei, China; 2 Third Clinical College of Yangtze University, Jingzhou, Hubei, China

**Keywords:** anterior cervical, internalfixation, morphometry, transpedicular cortical screw, visualization

## Abstract

**Background:**

The anterior transpedicular cortical screw technique for the subaxial cervical spine represents a novel surgical fixation method for treating cervical spondylosis. Due to the complex anatomical structure of the cervical spine, screw placement presents certain technical challenges. Research on screw placement-related parameters will facilitate the widespread clinical application of this technique.

**Objective:**

This study aimed to visualize and determine the optimal trajectory for placing cortical bone screws in the lower cervical spine (C3–C7) via an anterior approach, providing anatomical data for clinical application.

**Methods:**

Select 100 healthy adult volunteers (50 males and 50 females)and perform continuous CT scans of their cervical spine with a layer thickness of 0.625 mm and a spacing of 0 mm. Use MIMICS21.0 software to establish a three-dimensional digital model of the lower cervical spine, and create a hollow screw model with an outer diameter of 2.8 mm and an inner diameter of 0.5 mm. Visualize the placement of screws at different segments from anterior to posterior through the vertebral body and pedicle. Screw position was adjusted to maximize cortical purchase within the pedicle without breach. Parameters including the distances to the midline and superior endplate of entry points, and lateral/cephalad angles of the screws were measured. All measurements were conducted in MIMICS21.0 software, and the same data was measured by two people and averaged to compare the differences between male and female, left and right sides of the same indicator data.

**Results:**

The entry points of all four segments of the lower cervical spine are located on the same side of the pedicle. The distance between the entry points and the mid sagittal line, as well as the vertical distance to the upper endplate of the same vertebra, are between 1.96 mm–2.62 mm and 1.30 mm–1.93 mm, respectively, showing a gradual increase from C3 to C5 and a gradual decrease from C5 to C7. There is no statistically significant difference between the left and right sides of each segment or between males and females; All screws of the four segments of the lower cervical spine need to be tilted outward and upward, with tilt angles between 24.27°–39.42° and 16.60°–18.57°, respectively. The tilt angles also show a gradual increase from C3 to C5 and a gradual decrease from C5 to C7, with no statistically significant differences between the left and right sides of each segment or between males and females.

**Conclusion:**

It is feasible to insert cortical pedicle screws via the anterior cervical approach, maximizing screw purchase on cortical bone. Compared to conventional screws, this method may facilitate easier insertion and better plate conformity.

## 1 Introduction

Cervical pedicle screw fixation technique is a very effective method among many treatments for cervical diseases. In 2007, Koller et al. (1) proposed the anterior cervical pedicle screw internal fixation technique for treating cervical injuries and achieved good therapeutic effects. According to the anatomical research results related to cervical pedicle, it is feasible to insert 3.5 mm or thinner screws into it (2). Due to the complex anatomical structure and adjacent relationships of the cervical spine, there are significant variations between different individuals and different vertebrae within the same individual, and important structures such as vertebral arteries, spinal nerves, and spinal cord exist around it. Therefore, cervical pedicle screw fixation is considered a high-risk surgery (3). In clinical practice, in order to ensure the safety of screw placement, the basic method is to place screws along the anatomical axis of the pedicle, mainly relying on the gripping effect of the cancellous bone at the center of the pedicle to fix the screws. For some patients with osteoporosis or severe bone damage who require strong fixation, the failure rate of fixation such as screw loosening and screw withdrawal after surgery is relatively high (4). Compared with the weak purchase offered by cancellous bone, screws placed through a cortical trajectory provide superior uniaxial pull-out and torsional resistance. Theoretically, this enhances construct stability: the more cortical layers the screw traverses and the greater the cortical engagement, the stronger its biomechanical fixation becomes (5). Given the advantages of short surgical time, minimal surgical trauma, and fewer postoperative complications in anterior cervical decompression and fusion surgery, how to better combine the advantages of anterior pedicle cortical bone screws passing through multiple layers of cortex to ensure stability with a single anterior fixation is a clinical issue that needs to be studied, especially how to insert the needle to ensure more pedicle cortical occlusion of the screw. This study utilized Mimics digital software to perform 3D reconstruction based on CT datas of 100 healthy adult volunteers (50 males and 50 females) respectively. The principle was to visualize the placement of screws by biting more pedicle cortex without transmitting bone, and to conduct relevant morphological measurements to explore the morphological characteristics of screw placement, providing anatomical references for the design of corresponding fixation systems and clinical screw placement.

## 2 Materials and methods

### 2.1 Design

3D reconstructing by Mimics software, visualized screw implantating, and measuring of morphological parameters for anterior cervical pedicle screw fixation using cortical bone screws, observating of multiple specimens.

### 2.2 Time and location

The experiment was conducted from January 2024 to December 2024 at the Orthopedic Laboratory of Jingzhou Institute of Traditional Chinese Medicine.

### 2.3 Materials

#### 2.3.1 The research subjects

The research subjects were 100 adult volunteers (50 males and 50 females) aged 18–55 years old. They signed informed consent forms and obtained the approval of the hospital ethics committee to obtain the ethical batch number: Jingzhong Medicine Ethics Review Research (2023025).

Inclusion criteria: ① Age > 18 years old; ② Individuals with no previous symptoms, medical history, or surgical history related to spinal diseases; ③ The cervical CT scan shows normal cervical spine sequence, with no bone abnormalities such as fractures, severe bone hyperplasia or ligament ossification, abnormal cervical development, or cervical tumors.

Exclusion criteria: ① Age < 18 years old; ② Individuals with previous symptoms, medical history, or surgical history related to spinal diseases; ③ Cervical CT scan shows abnormal cervical spine sequence, or accompanied by bone abnormalities such as fractures, severe bone hyperplasia or ligament ossification, abnormal cervical development, cervical tumors, etc.

#### 2.3.2 Software and hardware equipment

64 row spiral CT (GE, USA), MIMICS 21.0 software (Materialise, Belgium), office computer (Lenovo, China). ent, cervical tumors, etc.

### 2.4 Methods

#### 2.4.1 Acquiring CT raw projection datas

CT scans were conducted at the Radiology Department of the Affiliated Hospital of Traditional Chinese and Western Medicine in our hospital. Fine and uninterrupted CT scans were performed on the entire cervical spine of 100 volunteers, covering the range from the distal occipital bone to T1. Scanning conditions: bone tissue window was selected, voltage was 120 kV, 65 Ma, pixel size was 0.43 mm, layer thickness was 0.625 mm, matrix was 512 × 512, and the scanned images were saved in DICOM format on a disc.

#### 2.4.2 Three-dimensional reconstructing the cervical spine

Involves placing the engraved disc into a computer, opening MIMICS software, reading CT data, determining the coordinate orientation, defining the bone threshold between 226 and 1,950 HU, selecting the modeling range of the entire cervical spine and enhancing the region, editing layer by layer, denoising to remove redundant data, and generating a three-dimensional model of the entire cervical spine through 3D calculation.

#### 2.4.3 Measuring anatomical parameters for different segment needle insertion parameters

##### 2.4.3.1 Measurement metrics

To elaborate on the measurement indicators and positioning standards for screw implantation, see Tables 1.

**Table 1:** Measurement metrics and positioning criteria for screw insertion.

Measurement metrics	Positioning standards
Open the distance to the left and right of the screw entry point	The distance between the projection point of the screw axis on the anterior wall of the vertebral body and the sagittal plane of the vertebral body
Distance in the up and down directions of the screw entry point	The distance between the projection point of the screw axis on the anterior wall of the vertebral body and the upper endplate of the vertebral body
Camber angle	The angle between the axis of the screw and the sagittal plane of the vertebral body
Upward tilt angle	The angle between the axis of the screw and the plane of the anterior wall of the vertebral body

##### 2.4.3.2 Measurement method

① Simulate screw placement: Display the constructed cervical spine model in 3D in MIMICS software, adjust the model orientation to display it in front and back positions; Design a hollow cylindrical simulated screw with an outer diameter of 2.8 mm, an inner diameter of 0.5 mm, and a length of 35 mm; Design a cylindrical body with a diameter of 0.5 mm and a length of 35 mm to be filled into the hollow cylinder, and display the two cylinders in different colors; Move the cylinder to the cervical model, enter and pass through the pedicle through the anterior cortex of the vertebral body, and tilt the screw towards the inner upper side inside the pedicle. Simulate inserting the screw on the other side in the same way.

② Screw path adjustment: Using the visualization and sectioning functions of MIMICS software, observe the position of simulated screws in the pedicle and vertebral body from the outside to the inside, and adjust the position of the simulated screws to the optimal state based on the principle of maximizing the purchase between the screws and the pedicle and upper cortex without penetrating the cortex. Adjust the screw on the other side in the same way.

**Figure 1:**
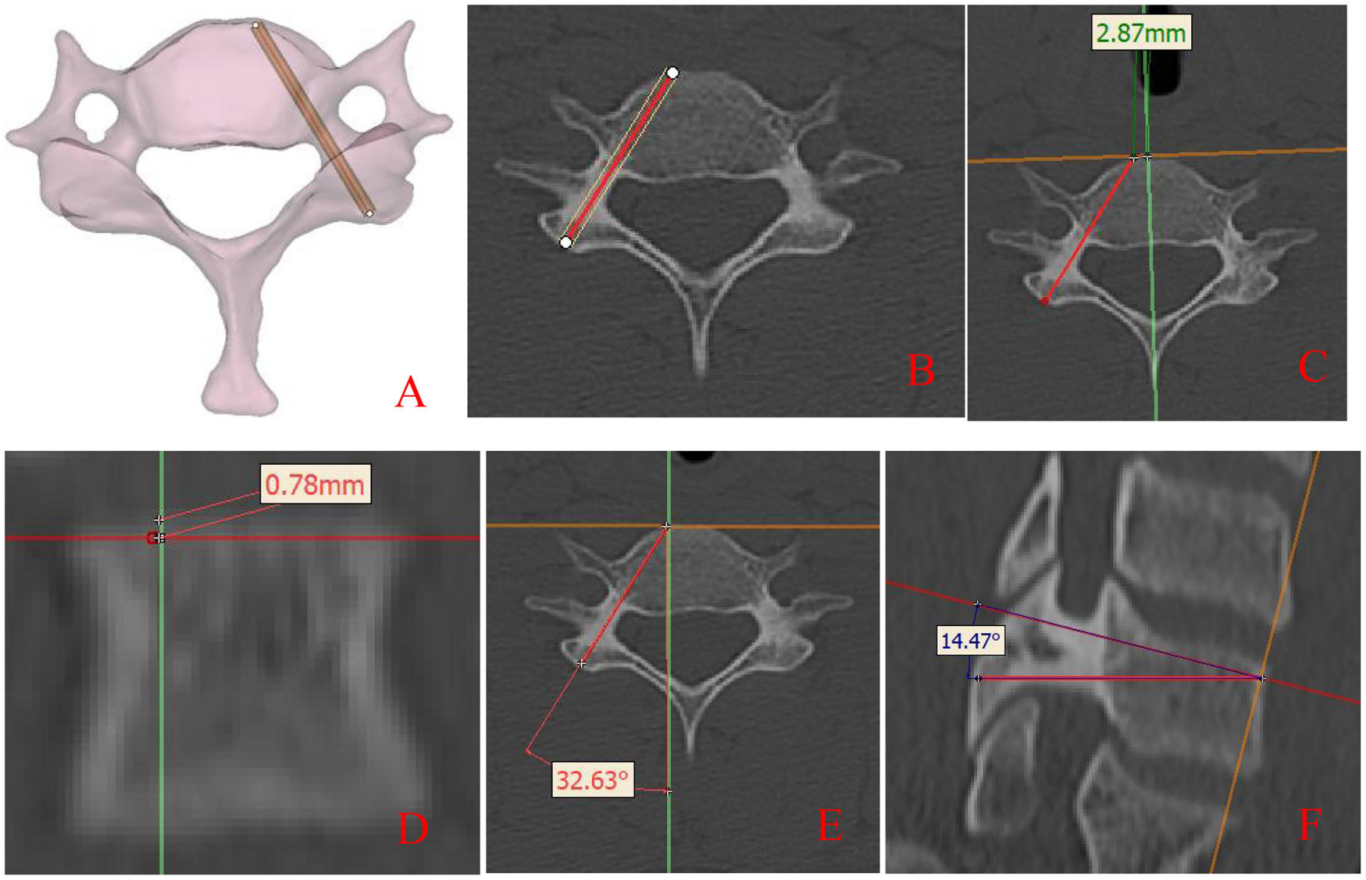
Localization and measurement **(A,B)** localization diagram; **(C,D)** distance measurement diagram; **(E,F)** angle measurement diagram.

③ Measurement method: Remove the empty cylindrical part of the simulated screw and leave the center thin cylinder as the central axis of the screw. Use the intersection point of the central axis and the front of the vertebral body as the needle insertion point of the screw. Use the straight-line distance and angle measurement tools on MIMICS software to measure on 2D and 3D images, respectively. The software automatically pops up the spacing and angle data of the object for direct reading. Each indicator data is measured by 2 people, and the average value is taken, with the data accurate to 0.01 mm and 0.01°. It should be explained that for the measurement of the distance between the left and right sides of the screw insertion point, if the axis of the screw is on the same side as the mid sagittal line, the measured value is a “positive” value, otherwise it is a “negative” value, as shown in Figure 1.

### 2.5 Statistical processing

The SPSS20.0 statistical software package was used for analysis, and the data was expressed as mean ± standard deviation $\left(\bar{x}\pm s\right)$. The data of each group were compared on the left and right sides, male and female respectively. Paired sample *t*-test (left and right sides) and Independent Samples *t*-test (male and female) were used for normally distributed data. *P* < 0.05 was considered statistically significant.

## 3 Result

### 3.1 Measurement parameters

Collect 50-male and 50-emale volunteers with a total of 100 normal cervical vertebrae, reconstruct 500 cervical vertebrae, measure 4,000 parameters, and analyze the 3D CT morphological measurement data of the lower cervical vertebrae (C3–C7).

### 3.2 Position of the screw entry point

As shown in Tables 2, 3, no statistically significant differences were observed (*P* > 0.05) in the overall mean of the distance between the left and right sides of the screw entry point and the distance between the upper and lower directions of the screw entry point between males and females, as well as between the left and right sides. From the measurement results, it can be seen that all the needle insertion points of the lower cervical spine are on the same side of the pedicle, and both the left and right lateral opening distances and the up-down orientation distances show a trend of gradually increased from C3 to C5, then decreased at C6 and C7.

**Table 2:** Parameters for screw entry point $\left(\bar{x}\pm s,\mathrm{mm}\right)$ .

Segment		Open the distance to the left and right of the screw entry point		Distance in the up and down directions of the screw entry point	
		Left	Right*	Left	Right*
C3	M (*n* = 50)	2.18 ± 0.46	1.62 ± 0.52	1.54 ± 0.30	1.48 ± 0.32
	F** (*n* = 50)	2.01 ± 0.31	2.03 ± 0.31	1.25 ± 0.23	1.28 ± 0.44
C4	M (*n* = 50)	2.13 ± 0.49	2.59 ± 0.65	1.75 ± 0.12	2.11 ± 0.48
	F** (*n* = 50)	2.26 ± 0.30	2.38 ± 0.23	1.42 ± 0.21	1.43 ± 0.24
C5	M (*n* = 50)	2.39 ± 0.64	2.94 ± 0.47	2.04 ± 0.27	1.60 ± 0.19
	F** (*n* = 50)	2.56 ± 0.22	2.58 ± 0.22	2.40 ± 0.48	1.69 ± 0.20
C6	M (*n* = 50)	2.15 ± 0.55	2.45 ± 0.23	1.73 ± 0.22	2.15 ± 0.60
	F** (*n* = 50)	2.04 ± 0.25	2.27 ± 0.34	1.38 ± 0.26	1.52 ± 0.35
C7	M (*n* = 50)	2.03 ± 0.48	2.30 ± 0.33	1.51 ± 0.30	1.80 ± 0.40
	F** (*n* = 50)	1.79 ± 0.26	1.78 ± 0.45	1.34 ± 0.33	1.36 ± 0.38

* *p* > 0.05.

** *p* > 0.05.

**Table 3:** Mean and range of the screw entry piont distance datas $\left(\bar{x}\pm s,\mathrm{mm}\right)$ .

Segment	Open the distance to the left and right of the screw entry point		Distance in the up and down directions of the screw entry point	
	Mean	Rang	Mean	Rang
C3	1.96	1.82–2.10	1.39	1.28–1.50
C4	2.34	2.19–2.49	1.68	1.55–1.81
C5	2.62	2.47–2.76	1.93	1.79–2.07
C6	1.98	2.10–2.35	1.70	1.54–1.85
C7	2.22	1.84–2.11	1.50	1.38–1.63

### 3.3 The screw direction

As shown in Tables 4 and 5, all vertebral body needle insertion directions need to be tilted outward and upward, and there is no significant difference in the outward and upward tilt angles between males and females, or between the left and right sides (*P* > 0.05). The inclination angle, whether outward or upward, gradually increases from C3 to C5 and then gradually decreases.

**Table 4:** Parameters for screw direction $\left(\bar{x}\pm s{,}^{\circ }\right)$ .

Segment		Camber angle		Upward tilt angle	
		Left	Right*	Left	Right*
C3	M (*n* = 50)	35.70 ± 2.48	34.52 ± 2.14	17.14 ± 1.32	16.41 ± 1.89
	F** (*n* = 50)	35.49 ± 1.42	34.13 ± 2.08	18.00 ± 0.49	18.12 ± 0.48
C4	M (*n* = 50)	37.52 ± 2.73	36.34 ± 3.63	17.18 ± 1.41	17.60 ± 3.01
	F** (*n* = 50)	38.22 ± 1.21	37.60 ± 1.68	18.39 ± 0.32	18.61 ± 0.71
C5	M (*n* = 50)	38.57 ± 2.72	38.38 ± 4.32	18.17 ± 1.04	17.85 ± 3.43
	F** (*n* = 50)	40.11 ± 1.09	40.59 ± 1.01	18.90 ± 0.34	19.36 ± 0.41
C6	M (*n* = 50)	36.67 ± 2.53	35.48 ± 1.71	17.19 ± 1.30	17.64 ± 3.46
	F** (*n* = 50)	36.97 ± 1.17	37.58 ± 1.49	18.26 ± 0.54	18.65 ± 0.69
C7	M (*n* = 50)	33.93 ± 2.75	33.75 ± 2.99	15.67 ± 1.90	16.10 ± 2.76
	F** (*n* = 50)	34.21 ± 1.54	35.21 ± 1.15	17.27 ± 0.65	17.35 ± 0.90

* *p* > 0.05.

** *p* > 0.05.

**Table 5:** Mean and range of the screw direction $\left(\bar{x}\pm s{,}^{\circ }\right)$.

Segment	Camber angle		Upward tilt angle	
	Mean	Rang	Mean	Rang
C3	34.96	34.29–35.63	17.42	16.99–17.85
C4	37.42	36.62–38.22	17.92	17.39–18.50
C5	39.42	38.52–40.29	18.57	17.98–19.16
C6	36.67	36.07–37.28	17.93	17.32–18.54
C7	34.27	33.56–34.99	16.60	16.01–17.19

## 4 Discussion

### 4.1 Advantages of anterior cervical pedicle screw fixation with cortical screws

With the change of human working and living habits and the gradual aggravation of aging, there are more and more cervical degeneration, and the incidence rate of cervical spondylosis is rising (6). From the perspective of the pathogenesis of cervical spondylosis, its compression mostly comes from the anterior part of the spinal canal, such as protruding intervertebral discs, ossified posterior longitudinal ligaments, and hypertrophic bone (7). Therefore, in clinical practice, surgical treatment for cervical spondylosis often involves anterior decompression combined with corresponding fixation to maintain the stability of the affected segment (8). There are two fixed methods: anterior fixation and posterior fixation. Although posterior laminectomy with decompression and internal fixation has been proven to be a simple, safe, and effective method of internal fixation with good stability, its principle is to expand the spinal canal and cause spinal cord drift, rather than direct decompression. For patients with cervical kyphosis or large protrusions, combined anterior and posterior surgery is sometimes required, which inevitably increases the surgical trauma and difficulty for patients (9). Therefore, simultaneous anterior decompression and anterior fixation are very important. The traditional anterior decompression and fusion surgery (ACDF) has been widely used in clinical practice. However, this method uses vertebral screws that only pass through the anterior cortex of the fixed vertebral body. Most of the screws are located in the cancellous bone, which is not stable enough for patients who require multi-stage fixation or osteoporosis. After surgery, the internal fixation becomes loose and the failure rate is high. In severe cases, revision surgery is required (10). To improve the stability of anterior surgical fixation, the anterior pedicle screw technique (APTS) has been proposed and clinically applied. Through biomechanical research, it has been found that the pull-out resistance of cervical anterior pedicle screws is 2.5 times that of anterior vertebral screws, while their mechanical properties are comparable to those of anterior posterior fixation (11). Currently, conventional screws with a diameter of 3.5–4.0 mm are widely used (12). Due to the complex anatomical structure of the cervical spine, which is adjacent to important tissue structures such as vertebral arteries, spinal cord, and nerve roots, The shortest distance between the cervical pedicle and the vertebral artery ranges from 1.5 mm to 2.5 mm (13), while that to the nerve root spans 1.0–3.0 mm (14), the medial wall abuts the dural sac (15). Any breach of the pedicle cortex poses a risk of vascular and neurological injury. In order to reduce the risk of screw insertion, it is currently required that the screw be inserted along the central axis of the pedicle. As the transverse diameter of the cervical pedicle is significantly smaller than the longitudinal diameter, even with such insertion, there is a high risk of screw penetration. Previous studies have found that finer diameter cortical screws, as long as they are placed accurately and purchase as much pedicle cortex as possible during placement, will provide more stable fixation (16). Therefore, anterior cervical pedicle cortical screws have also been applied in clinical practice, but they are different from conventional screws in that they have a thinner diameter and require more cortical occlusion. Therefore, they cannot enter the pedicle along the central axis, which requires their entry point position and internal/external and vertical tilt angles to be different from conventional screws. Previous studies have mainly focused on conventional screws (17), and there have been no reports specifically on cortical screws.

### 4.2 Analysis of screw placement parameters

Through previous morphological observations of the cervical pedicle and other studies, the placement parameters of each vertebra are particularly important to ensure accurate screw placement, including the positioning of the screw insertion point and the angle of inclination of the screw inside and outside (18). This study mainly measured four data points: the distance of the screw tail from the upper endplate in the transverse section, the distance from the mid sagittal line in the sagittal plane, and the angle of the screw axis deviation from the anterior posterior midline and the upper and lower planes. From the statistical results, there is no significant difference in the above data between the left and right sides of the same vertebra and between male and female genders. Therefore, gender and left-right differences do not need to be considered when clinical screw placement. From the position relationship between the screw insertion point and the mid sagittal line, it can be seen that all the screw insertion points of the lower cervical spine are located on the same side of the pedicle, which is different from the conclusion of other scholars' studies on conventional pedicle screws (19, 20). The reason for this may be related to the thinner diameter of cortical screws and the different screw channels compared to conventional screws. From this conclusion, it can be seen that cortical screws combined with steel plates for fixation are more convenient. Since the screws on the left and right sides do not intersect in the vertebral body, the screws on both sides can be in the same horizontal plane, making the matching between screws and steel plates simpler and the fixation effect better; From the distance of deviation from the midline of the screw entry point, it gradually increases from C3 to C5, and then gradually decreases again at C7, with an absolute value between 1.96 mm and 2.62 mm. This is different from the measurement values of conventional screws studied by previous scholars (21), and the reason may still be the difference in the screw path. From the measured results, it can be seen that even considering the factor that the diameter of the cortical screw tail cap is larger than the diameter of the screw body, in clinical cortical screw placement, the left and right screw tail caps will not overlap in front of the vertebral body and affect the fixation effect of the screw; From the distance between the screw insertion point and the upper endplate of the same vertebral body, it also shows a pattern of gradually increasing from C3 to C5, and then gradually decreasing from C7. The average distance is between 1.30 mm and 1.93 mm. From this result, it can be seen that when inserting a 2.8 mm diameter screw, it ensures maximum occlusion of the cortex without penetrating the upper endplate; From the perspective of screw insertion, all lower cervical vertebrae should be inclined outward and upward, but the angle is smaller than that of conventional screws (22, 23). The smaller the angle, the better the match between the screw, especially the screw cap, and the steel plate may be. From the overall measurement data, it is feasible to insert cortical bone screws through anterior approach to purchase as much cortical bone as possible, and compared to conventional screws, it can better place the screws and match the steel plate.

### 4.3 Advantages of visualizing screw trajectory research

The advantages of visualizing screw trajectories in interactive medical image control system (MIMICS) software have been proven to be scientifically effective in constructing three-dimensional images of cervical spine bones for the study of relevant parameters of screw trajectories (24). It has the advantages of visualizing screw trajectories, combining two-dimensional and three-dimensional positioning, and digitizing the reading of positioning parameters. Compared with cadaveric specimens, the research results are more reliable. In the early stage of this study, measurements were taken on the pedicle cortex of CT specimens of the lower cervical spine. The results showed that the inner and outer cortex of the pedicle were thicker than upper and lower walls of each segment. Therefore, it is believed that in order to use screws to purchase more cortex, it should be biased towards the inner and upper parts. Due to the fact that the inner side is the spinal canal and contains the spinal cord, screws cannot penetrate the inner cortex. In the study, we applied the visualization function of Mimics software to view the relationship between the screw and the spinal canal wall from multiple angles when adjusting the screw position, in order to ensure that the screw purchases as much cortex as possible without penetrating the spinal canal, and to ensure the guiding significance of the measured data for clinical practice. To facilitate data measurement, in our study, two cylinders with different thicknesses were used to simulate screw placement. A large cylinder with a diameter of 2.8 mm was used to simulate the thickness of the screw, and a small cylinder with a middle diameter of 0.5 mm was used to simulate the central axis of the screw. After adjusting the screw position, the large cylinder was removed and the small cylinder was left for data measurement, making the data more accurate; In the research, the interactive positioning of two-dimensional and three-dimensional images using software is fully utilized to more accurately determine the position of screws and measure relevant parameters; Using normal CT data of the human body to construct a digital model, the data source is convenient, and there is no need to worry about the model being damaged and it can be reused repeatedly (25). In summary, these advantages cannot be met by macroscopic specimens, therefore the conclusions drawn in this study have strong scientific validity and high reliability.

### 4.4 Shortcomings of this study

Firstly, this study is still a single center small sample study. In order to further enhance the scientific validity and representativeness of the research results, multi center and large sample studies are needed in the later stage; Secondly, this study only measured four indicators, including the position and inclination angle of the screw insertion point, and further verification is needed to determine whether the data is sufficient; Third, this study selected screws with a smaller diameter (2.8 mm) and denser thread pitch for screw placement simulation, and their mechanical stability remains to be further investigated. Fourth, the guiding significance of the experimental results for clinical practice still needs to be clinically tested, in order to have more relevant clinical feedback in the later stage, further deepen the experiment, better integrate the experiment with clinical practice, and produce more valuable results.

## 5 Conclusion

The anterior cervical pedicle cortical screw technique is a surgical fixation method for treating cervical spondylosis that remains to be further investigated. Through visualized screw canal research, it was found that its screw placement method is different from conventional pedicle screws. In clinical operation, for different lesion segments, while clarifying basic indicators such as point position and deviation angle, individualized principles should be followed to improve preoperative imaging examinations, fully analyze the anatomical structure characteristics of the patient's lesion site, and develop personalized surgical screw placement plans. At the same time, advanced equipment such as intraoperative fluoroscopy, surgical navigation templates, and surgical robots should be used to ensure surgical safety.

## Data Availability

The original contributions presented in the study are included in the article/Supplementary Material, further inquiries can be directed to the corresponding author.
